# HBx interacts with the host YBX3 protein and up-regulates its expression to mediate efficient Hepatitis B viral replication

**DOI:** 10.3389/fcimb.2026.1732356

**Published:** 2026-05-22

**Authors:** Mel Veen Boo, Jackie Chu, ZiJie Lim, Som Mohanlal Patwa, Wan Jin Hong, Seng Gee Lim, Jayantha Gunaratne, Yee Joo Tan

**Affiliations:** 1 Infectious Diseases Translational Research Programme and Department of Microbiology and Immunology, Yong Loo Lin School of Medicine, National University Health System, National University of Singapore, Singapore, Singapore; 2 Institute of Molecular and Cell Biology (IMCB), Agency for Science, Technology and Research, (ASTAR), Singapore, Singapore; 3 Division of Gastroenterology and Hepatology, Department of Medicine, National University Hospital, University Medicine Cluster, National University Health System, Singapore, Singapore; 4 Department of Anatomy, Yong Loo Lin School of Medicine, National University of Singapore, Singapore, Singapore

**Keywords:** antiviral therapeutic development, HBx, proviral host factor, viral replication, YBX3

## Abstract

**Background:**

Chronic Hepatitis B (CHB) remains a global health challenge, and identifying the precise viral-host interactions that sustain the viral life cycle is essential for developing next-generation therapeutic strategies. In this study, we identify the host factor YBX3 (Y-box binding protein 3) as a critical requirement for efficient Hepatitis B Virus (HBV) replication.

**Methods:**

Using immunoprecipitation-coupled mass spectrometry, we identified YBX3 as a novel interactor of the HBV regulatory protein, HBx. This interaction was validated via co-immunoprecipitation. The functional role of YBX3 was characterized using CRISPR/Cas9-mediated knockout in HepG2-hNTCP cells, followed by genetic rescue experiments. Viral fitness was assessed by quantifying cccDNA, pgRNA, HBsAg, HBeAg, and extracellular HBV DNA. Subcellular fractionation was employed to determine the distribution of YBX3 and HBx.

**Results:**

YBX3 interacts specifically with HBx via its C-terminal domain. Genetic ablation of YBX3 drastically impaired HBV replication, resulting in a significant reduction in viral DNA, HBeAg, and HBsAg, a phenotype significantly reversed by YBX3 re-expression. Subcellular fractionation confirmed that both proteins are predominantly nuclear and that HBx expression upregulates YBX3 abundance in both nuclear and cytoplasmic fractions. YBX3 mRNA and protein levels were significantly upregulated during active HBV infection, suggesting a proviral feedback loop.

**Conclusion:**

YBX3 is a novel proviral host factor that drives efficient HBV replication by binding to HBx. This interaction likely supports the crucial nuclear functions of HBx that are vital for efficient viral replication. These findings establish YBX3 as a compelling new target for antiviral therapeutic development.

## Introduction

Chronic Hepatitis B (CHB) remains a significant global health challenge, affecting over 250 million individuals worldwide and potentially resulting in severe liver complications including fibrosis, cirrhosis, and hepatocellular carcinoma (Lampertico et al., 2017). The Hepatitis B virus (HBV) lifecycle is initiated when viral particles bind to the sodium taurocholate co-transporting polypeptide (NTCP) receptor on hepatocyte membranes, and hence the entry of the virus is facilitated (Yan et al., 2012). Following internalization, relaxed circular double-stranded DNA (rcDNA) is released and transported to the nucleus, where it either integrates into the host genome or converts to covalently closed circular DNA (cccDNA) (Shafritz, 1982; Guo et al., 2007). This cccDNA associates with host histones to form a stable minichromosome serving as the transcriptional template for viral proteins including HBV surface antigen (HBsAg) and HBV e-antigen (HBeAg), as well as progeny virions (Bock et al., 1994; Levrero et al., 2009). The persistent presence of cccDNA in infected cells presents a significant barrier to achieving sterilizing cure. Additionally, the continuous secretion of HBsAg and HBeAg suppresses both innate and adaptive immune responses, hindering viral clearance (Bertoletti and Boni, 2021). Current therapeutic interventions for CHB predominantly comprise interferon alpha (IFN-α) and nucleoside/nucleotide analogues (NAs). While these modalities demonstrate modest efficacy in attenuating disease progression in the short term, their clinical utility is substantially constrained by the cumulative adverse event profile associated with prolonged administration, necessitating the development of alternative therapeutic strategies with improved safety profiles and enhanced curative potential. These limitations present the urgent need for innovative approaches to identify next-generation anti-HBV targets and develop complementary therapeutic agents.

The HBV is a partially double-stranded DNA pathogen of the Hepadnaviridae family, and it displays pronounced hepatotropism. The virus possesses a remarkably economical 3.2-kB genome encoding four fundamental genes: polymerase (*P*), core (*C*), surface (*S*), and *X*. Among these, the 154-amino acid (17-kD) HBx protein merits particular attention, as paramount evidence demonstrates its indispensability for productive infection in human liver chimeric murine models and HepaRG cell systems (Tsuge et al., 2010; Lucifora et al., 2011), while concurrently facilitating optimal viral replication in plasmid-based experimental paradigms (Melegari et al., 1998; Bouchard et al., 2003). This regulatory protein exhibits remarkable functional versatility, including transactivation of both viral and cellular promoters (Murakami, 2001), interaction with cccDNA to orchestrate epigenetic modifications (Koumbi and Karayiannis, 2016), and targeted degradation of host restriction factors (Minor et al., 2020). While exhibiting significant oncogenic potential, HBx is crucially important for viral replication, encompassing transcriptional regulators (YY1; Shen et al., 2020), protein processing factors (SIRT2; Cheng et al., 2018), DNA damage response proteins (DDB1; Leupin et al., 2005), and cellular trafficking components (VCPIP1; Kang et al., 2024). Elucidating these specific host factor interactions is crucial for identifying novel targets for antiviral intervention. Therefore, Host Targeting Agents (HTAs) have emerged as a particularly compelling therapeutic strategy in recent years, primarily because host proteins exhibit substantially reduced mutational plasticity under selective pressure compared to viral targets. While certain host-directed therapies have achieved demonstrable success (as reviewed in Mohd-Ismail et al., 2019), exemplified by the NTCP-targeting entry inhibitor myrcludex B (now clinically approved for Hepatitis D virus infection) (Bogomolov et al., 2016), the development of HTAs against HBV remains notably constrained. This therapeutic gap predominantly reflects insufficient mechanistic understanding of critical virus-host molecular interactions, particularly those governing the initial phases of viral infection.

To expand the repertoire of cellular proteins interacting with HBx, we performed an initial screen utilizing immunoprecipitation (IP) coupled with mass spectrometry (MS). This analysis identified host protein YBX3, which is a member of the Y-box binding (YBX) protein family, as a potential HBx interactor. Co-immunoprecipitation (co-IP) experiments definitively established an interaction between the host protein YBX3 with HBx protein via a C-terminal domain that is not conserved in YBX1. Furthermore, functional validation experiments revealed that HBV replication is greatly reduced in the absence of YBX3 and suggest that the YBX3 pathway represents a promising novel target for anti-HBV drug discovery.

## Materials and methods

### Cell lines

293FT and Cos-7 cells (Thermo Fisher) were cultured in DMEM with 10% heat-inactivated FBS. The parental HepG2, HepG2-hNTCP and Hep38.7 tet-off cells (Ogura et al., 2014) were maintained in complete DMEM/F-12 (Gibco) supplemented with 5% heat-inactivated FBS, 2 mM L-glutamine, 100 U/mL penicillin/streptomycin, 0.1 mM NEAA, and 5 µg/mL insulin (Sigma). Hep38.7 cells additionally contained 0.1 mg/mL Doxycycline (Clontech) to suppress HBV expression. All cells were cultured at 37 °C with 5% CO_2_.

### Transfection, immunoprecipiation, In-gel protein digestion and MS analysis

HepG2-hNTCP cells were transiently transfected with polyethylenimine (PEI) (constructs in **Supplementary Table 1**). Lysates, prepared with IP lysis buffer (50 mM Tris–HCl pH 7.2, 10 mM NaCl, 1% NP-40, protease inhibitors), were pre-clarified with Protein G agarose. Clarified lysates were incubated with anti-Flag M2 antibody (Sigma) overnight at 4 °C. Proteins eluted from Flag antibody beads were subjected to short SDS-PAGE after adding Laemmli buffer with β-mercaptoethanol (BME). Gels were stained with InstantBlue (Abcam), destained, and bands excised. In-gel tryptic digestion followed published procedures (Shevchenko et al., 2006). Lyophilized tryptic peptides were reconstituted in 0.1% FA and analyzed by Q Exactive Plus coupled to Proxeon n1000 nanoLC. Peptide elution used a 2-90% Buffer B gradient over 60 mins (200 nL/min). MS settings: survey scan (300–1400 m/z, 140, 000 resolution, AGC 3e6, 50 ms max IT), top 20 peptide ions fragmented by CID (NCE 35%, scan 200–2000 m/z, 17, 500 resolution, AGC 3e6, 1.2 m/z isolation window). Raw data were analyzed with PD2.2, using Sequest HT against a Uniprot protein database (104508 proteins). Search parameters: 10 ppm Precursor Mass Tolerance, 0.06 Da Fragment Mass Tolerance. Dynamic modification: +15.995 Da (M); static modification: +57.021 (C). LFQ quantification used Minora Feature Detector and Precursor Ions Quantifier.

### Transient transfection followed by co-IP

293FT cells were transiently transfected with plasmids using PEI (**Supplementary Table 1**). After 48 h, cells were lysed in RIPA buffer (50 mM Tris-HCl pH 8.0, 150 mM NaCl, 0.5% NP-40, 0.5% sodium deoxycholate) with protease inhibitor cocktail (Roche) for 15 mins at 4 °C. Lysates were centrifuged (14, 000 x g, 10 mins, 4 °C), aliquots of clarified lysates were used for Western blot (as ‘input’) or incubated overnight with anti-FLAG M2 beads (Sigma). Beads were extensively washed with binding buffer (RIPA without inhibitors), then boiled in 1X Laemmli buffer with BME to elute proteins for Western blot analysis.

### HBV infection

HBV was purified from stable Hep38.7 tet-off cells. HBV infection was performed according to (Lim et al., 2022). Briefly, cells were pre-incubated for 24 h in complete medium with 2% FBS and 3% DMSO. Infection was at MOI 3000 in fresh DMEM/F12 containing 4% PEG8000, 3% DMSO, and 2% FBS for 24h. Mock infections excluded HBV. After 24 h, cells were washed 3x with PBS and replaced with post-infection DMEM (2% FBS, 1% DMSO), changed every 2–4 days. Cells and corresponding culture media were harvested simultaneously at experimental endpoints.

### siRNA-mediated transient silencing

To transiently silence YBX3 expression, HepG2-hNTCP cells were seeded in 24-well plates at a density of 3 x 10^5^ cells/well and transfected with 20 nM of YBX3-targeting siRNA (Dharmacon) or a scrambled siRNA using Lipofectamine RNAiMAX (Invitrogen) according to the manufacturer’s instructions. At 24 h post-transfection, the media was replaced with complete medium supplemented with 2% FBS and 3% DMSO. The following steps were performed according to the methods in “HBV infection” section. Both the culture supernatant and cells were harvested at 4 or 6 d.p.i for further analysis.

### Transfection with full HBV genome of genotype C

Both parental HepG2 cells and HepG2-hNTCP-KO cells were seeded in 24-well plates at a density of 3 x 10^5^ cells/well. After 24 hours, cells were transfected with 0.5 µg of the Genotype C HBV plasmid (**Supplementary Table 1**) using Lipofectamine 3000 (Invitrogen) according to the manufacturer’s instructions. At 24 h post-transfection, cells were washed with PBS and replaced with fresh medium supplemented with 1% DMSO. Both the culture supernatant and cells were harvested 7 days post-transfection for further analysis.

### Subcellular fractionation

HepG2-hNTCP cells (transfected with empty vector/FLAG-HBx, or mock/HBV-infected at MOI 3000) were harvested in PBS. Subcellular fractionation into nuclear and cytosolic fractions was performed using a Nuclear/Cytosolic Fractionation Kit (Cell Biolabs) per manufacturer’s protocol. Fractions were analyzed by Western blotting for YBX3, HBx expression, and localization. Protein band intensities of YBX3 in the nuclear and cytosolic fractions were quantified separately. For each compartment, the intensity values were normalized to their respective compartmental markers and then expressed as a ratio relative to the empty vector/mock-infected control.

### SDS-PAGE and western blot analysis

Proteins were separated by SDS-PAGE (homemade Tris-Glycine gels, 8-12% based on MW). Proteins were wet-transferred to nitrocellulose membranes (Bio-Rad system). Membranes were blocked with 5% milk in 1X PBST, then incubated with primary antibodies (**Supplementary Table 2**) diluted in 1X PBS with 0.25% Tween 20. Membranes were washed with PBST between all antibody incubation steps. Protein detection used Pico Super Signal ECL substrate (Pierce) and images captured with an iBright Imaging system (Thermo Fisher). For quantitative assessment of YBX3 abundance, densitometric analysis (ImageJ) was performed. YBX3 band intensity was normalized to β-actin, and these values were further normalized against corresponding mock-treated samples to determine relative fold changes. All uncropped blots could be found in **Supplementary Figures 1**-**3**.

### Nucleic acid extraction and cDNA synthesis

Total RNA was extracted from cells using RNeasy Mini Kit (Qiagen). 250–400 ng of total RNA was reverse transcribed using iScript Select cDNA synthesis kit (Biorad) per manufacturer’s protocol. 10 ng cDNA was used for qPCR. Secreted HBV DNA was extracted from 200 μl of clarified culture supernatant using the QIAamp DNA Mini Kit (Qiagen) according to the manufacturer’s instructions. To ensure the complete dissociation of the viral polymerase from the encapsidated DNA, samples were incubated with Proteinase K at 56 °C for 1 h prior to column loading. Extracted DNA was eluted in nuclease-free water and quantified by real-time PCR. For intracellular cccDNA, HepG2-hNTCP cells were harvested at 7 d.p.i. To specifically enrich for the cccDNA species, we employed a modified alkaline lysis-based protocol using the FavorPrep™ Plasmid Extraction Mini Kit (Favorgen). Briefly, cells were lysed in an alkaline environment to denature chromosomal DNA, followed by neutralization to precipitate high-molecular-weight genomic DNA and cellular proteins. The resulting supernatant was purified via silica-gel membrane centrifugation. To ensure the removal of non-episomal HBV DNA, 150 ng of the extracted DNA was treated with T5 Exonuclease (New England Biolabs, 10 U/μL) for 1 h at 37 °C. This exonuclease specifically digests linear DNA and relaxed circular DNA (rcDNA) containing nicks or gaps, leaving the covalently closed circular DNA (cccDNA) intact. The reaction was terminated via heat inactivation at 70 °C for 30 min prior to qPCR.

### Real-time quantitative PCR

mRNA expression of *YBX3* and *GAPDH* (housekeeping gene) were quantified by qPCR. Reactions (10 µL) used PowerTrack SYBR Green Mastermix (Applied Biosystem) on a StepOne Plus Real-Time PCR System (Applied Biosystem), performed in duplicate for each biological replicate. Primer details in **Supplementary Table 3**. Relative gene expression was calculated using the 2^−ΔΔCt^ method, normalized to *GAPDH*. Relative fold changes for *YBX3* transcripts were normalized to their respective mock-infected controls. Extracellular HBV DNA, intracellular pgRNA, and cccDNA was amplified using forward (5′-GGGGCGCACCTCTCTTTA-3′) and reverse (5′-AGGCACAGCTTGGAGGC-3′) primers. To further confirm the specificity of the cccDNA quantification, results were independently validated using a pair of gap-spanning primers (F: 5′- GTGGTTATCCTGCGTTGAT-3′; R: 5′- GAGCTGAGGCGGTATCT-3′) to exclusively amplify the circularized viral genome. Absolute copy number for all viral targets were determined by standard curve.

### HBeAg and HBsAg ELISA

HBeAg ELISA was performed on 200 µL of culture medium (mock/HBV-infected) using the QuickTiter Hepatitis “e” Antigen ELISA kit (Cell Biolabs) per manufacturer’s protocol. HBsAg ELISA was performed on 20 µL of culture medium (mock/HBV-infected) using the Monolisa HBsAg ULTRA kit (Bio-Rad) per manufacturer’s protocol. Absolute concentrations (ng/ml) were determined using serial dilutions of recombinant HBeAg and HBsAg as standards.

### CRISPR-Cas9 plasmid construction

To generate a stable knockout of YBX3, two distinct sgRNAs targeting exon 1 and exon 10 of the human *YBX3* gene were designed using CHOPCHOP (https://chopchop.cbu.uib.no/). The sgRNA sequences were selected based on high predicted on-target efficiency and minimal predicted off-target activity. The specific sgRNA sequences used were: sgRNA 1: 5’-CCCCGCGCCCAAGAGCCCGG-3’ and sgRNA 2: 5’-CCTCTCCAGTTTCAGCAACCGCT-3’. For cloning, the sense and antisense oligonucleotides for each sgRNA were first phosphorylated and annealed by incubating the reaction mixture at 37 °C for 30 minutes, followed by heating to 95 °C for 5 minutes, and then slowly cooling to room temperature. The annealed sgRNA oligonucleotides were then ligated into the LentiCRISPRv2-GFP lentiviral expression vector, which had been linearized by digestion with the BsmBI restriction enzyme (New England Biolabs). Ligation was performed using T4 DNA Ligase (Thermo Fisher). Ligated plasmids were transformed into DH5α competent cells via heat shock. Colonies were selected and sequenced to confirm the insertion of sgRNA. Plasmid DNA was isolated using a Plasmid Midi Prep Kit (Qiagen).

### Lentivirus production using 3^rd^ generation lentiviral plasmids

293FT cells (90% confluence) were transfected with a third-generation lentiviral packaging system (**Supplementary Table 1**) using PEI. The medium was replaced 24 hours post-transfection, and the lentiviral supernatant was harvested 24 hours later (48 hours post-transfection). The supernatant was then filtered (0.45 µm).

### Generation of YBX3-KO HepG2-hNTCP cells

Wild-type HepG2-hNTCP cells were transduced with lentiviral supernatant containing 6 µg/mL polybrene. Successful transduction was assessed by GFP expression at 48 h. Cells were trypsinized, filtered (0.45 µm), and GFP-positive single cells were sorted into 96-well plates using a FACS sorter (BD FACS Aria). Clonal populations were expanded and validated for YBX3 knockout by Western blotting.

### Cell fitness

Cell viability was assessed using the Celltiter-Blue (Promega) assay. WT and *YBX3*^(-/-)^ HepG2-hNTCP cells were seeded in 96-well plates and monitored over 4 days. At the indicated time points, CellTiter-Blue reagent was added to each well and incubated for 2 h at 37 °C. The absorbance was measured at 570 nm and 600 nm using a microplate reader (Tecan). Cell viability was expressed as a percentage relative to the WT control group.

### NTCP expression

NTCP protein levels were assessed by Western blot. Total protein was extracted from WT and YBX3-KO cells, and 20 µg of lysate was resolved by SDS-PAGE and probed with an anti-NTCP antibody (**Supplementary Table 2**).

### Functional rescue experiment

*YBX3*^(-/-)^ HepG2-hNTCP cells were seeded at 70% confluence and transfected with 1 µg of either an empty vector (EV) or a FLAG-YBX3 expression plasmid using Lipofectamine 3000 (Thermo Fisher Scientific). The transfection medium was replaced 6 hours post-transfection with fresh complete medium supplemented with 3% DMSO. Twenty-four hours post-transfection, cells were infected with HBV as described above. Culture medium and cell lysates were harvested to quantify viral markers including extracellular eDNA, HBeAg, HBsAg, and intracellular pgRNA at 5 d.p.i. (6 days post-transfection).

### Statistical analysis

All assays were repeated three times. Specifically, each experiment utilized a separate biological sample (a different passage of parental HepG2/HepG2-hNTCP cells) for each condition, processed on three separate occasions. For mRNA expression studies, HBeAg and HBsAg quantification, each biological sample was analyzed in technical duplicates. Statistical significance was determined using GraphPad Prism 8.0. For experiments comparing two independent groups, a two-tailed Student’s t-test was performed. For comparisons involving multiple groups across different time points, a two-way ANOVA followed by a Tukey’s *post-hoc* test was performed to determine which specific groups were significantly different from each other. Data are presented as the mean + SEM. Asterisks were used to indicate statistical significance for the t-test: ∗∗*p* < 0.01, ∗∗∗*p* < 0.001, and ∗∗∗∗*p* < 0.0001. Different letters indicate statistical significance (*p* < 0.05), with groups not sharing the same letter considered significantly different.

## Results

### Identification of the host YBX as one of the potential interactors with viral HBx protein

An IP-MS approach in HepG2-hNTCP cells identified 38 host proteins interacting with HBx (HBx vs. empty vector; **Supplementary Table 4**). Given that HBx regulates key viral pathways in the nucleus (Keasler et al., 2009), it is noted that 15 interactors are known to have nuclear localization (**Table 1**). Notably, Heat shock cognate 71 kDa Protein 8 (HSPA8) and Reptin (RUVBL2) exhibited exceptionally high confidence of interaction, indicated by their substantial percentage coverage (62.50% and 43.19%, respectively). While these top two novel HBx interactors are highly significant and merit dedicated functional analysis in future studies, the primary focus of the present investigation was to elucidate the role of the third-ranked candidate, YBX3, which is a member belonging to the YBX family, with a percentage coverage of 33.60%. We prioritized YBX3 due to its less-explored connection to HBV replication. Furthermore, another member of this family, YBX1 was shown to interact with HBV by promoting cccDNA formation, which is the stable template for HBV gene transcription and crucial for viral persistence (Verrier et al., 2022). Thus, the interaction of YBX3 with HBx was further investigated and compared with YBX1’s proviral mechanism.

**Table 1:** Potential nuclear host interactors of HBx identified by IP-MS.

Protein (abbreviation)	Percentage coverage (%)
Heat shock cognate 71 kDa (HSPA8)	62.50
Reptin (RUVBL2)	43.19
Y-box -binding protein 3 (YBX3)	33.60
U2 small nuclear ribonucleoprotein B (SNRPB)	23.11
Nuclear cap-binding protein subunit 2 (NCBP2)	12.18
F-box-like/WD repeat-containing protein TBL1XR1 (TBL1XR1)	7.39
Armadillo repeat-containing X-linked protein 3 (ARMCX3)	7.12
Guanine nucleotide-binding protein-like 3 (GNL3)	7.10
Calcineurin B homologous protein 1 (CHP1)	6.15
Large proline-rich protein BAG6 (BAG6)	4.48
Copine-1 (CPNE1)	3.72
Zinc finger protein 207 (ZNF207)	2.72
ATP-dependent RNA helicase DDX18 (DDX18)	1.34
Activating molecule in BECN1-regulated autophagy protein 1 (AMBRA1)	1.08
Neuroblast differentiation-associated protein AHNAK (AHNAK)	0.20

These potential host interactors were selected based on positive log-fold change (logFC > 0) and a p-value less than 0.05 (*p* < 0.05), indicating statistically significant enrichment. Percentage coverage was calculated based on the ratio of the total number of amino acid residues identified by the peptides to the total number of amino acid residues in the full-length protein.

### The binding of YBX3 to HBx occurred through its CTD region

**Figure 1:**
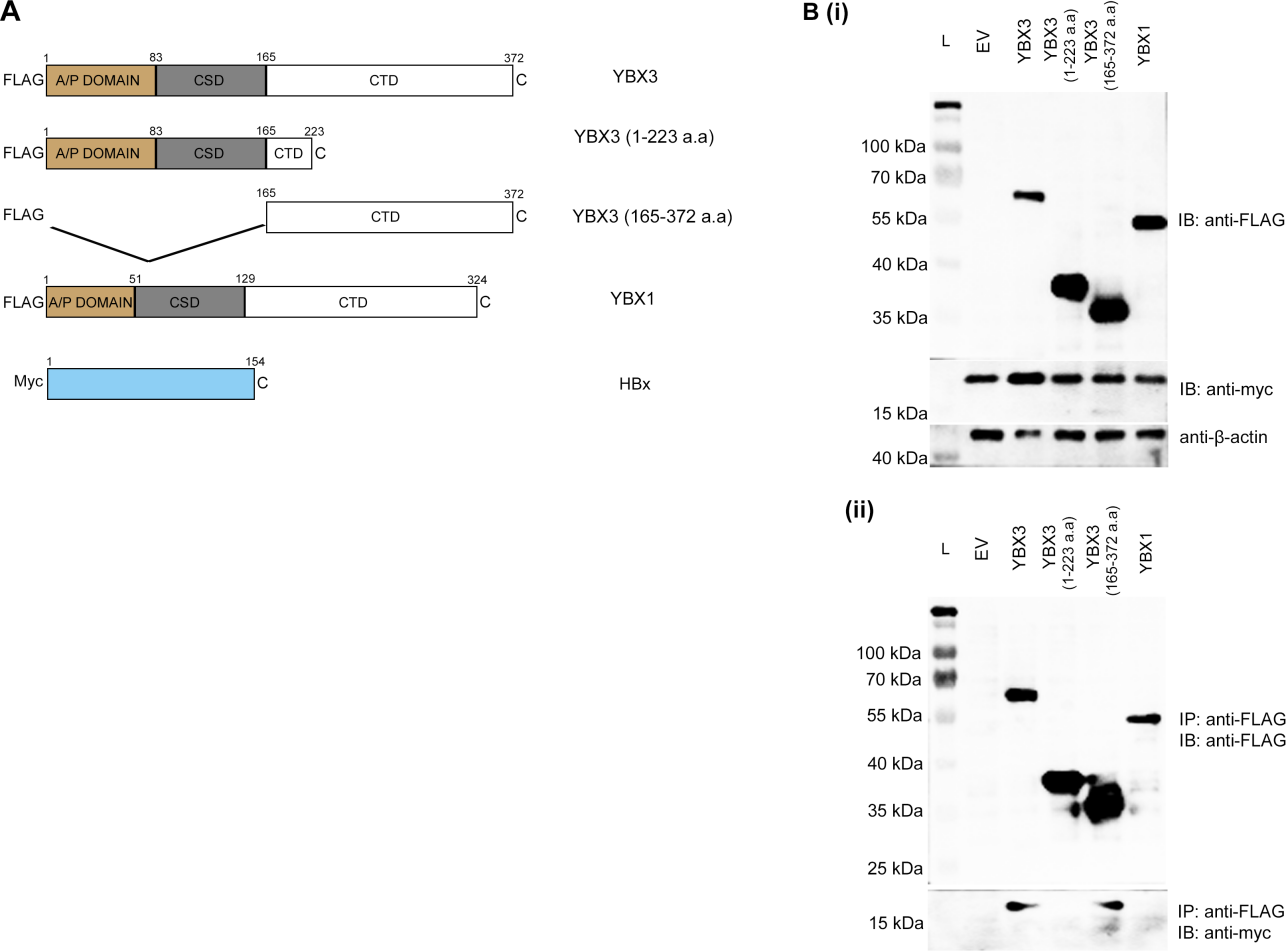
HBx interacts exclusively with the C-terminal domain of YBX3. **(A)** Schematic diagram of plasmids used in Co-IP experiments, including full-length YBX3, YBX1 and HBx, and the truncated mutants of YBX3 for domain mapping. (**B, i**) This served as the ‘input’ purpose to confirm the expression levels of the FLAG-tagged proteins and myc-tagged HBx across all experimental conditions. The lysates of the FLAG-tagged plasmids and myc-tagged HBx were detected with anti-FLAG and anti-myc antibodies, respectively. β-actin served as a loading control. (**B, ii**) From each lysate that contains one specific FLAG-tagged protein and co-expressed myc-tagged HBx, the respective FLAG-tagged protein was immunoprecipitated using FLAG antibody beads. The blot was then probed with anti-FLAG and anti-myc antibodies to assess the presence of the immunoprecipitated FLAG-tagged protein and the co-immunoprecipitated myc-tagged HBx, respectively. L represents protein ladder; EV represents empty vector.

To identify the region on YBX3 mediating its interaction with HBx, 293FT cells were co-transfected with plasmids expressing myc-tagged HBx along with either FLAG-tagged YBX1 or FLAG-tagged YBX3. Western blotting confirmed the expression of these overexpressed FLAG-tagged proteins, revealing prominent bands at approximately 65 kDa for YBX3, and 55 kDa for YBX1. Subsequent detection of myc-tagged HBx revealed an interaction with FLAG-tagged YBX3, but not with FLAG-tagged YBX1. Analysis using two truncated YBX3 mutants [YBX3 (1–223 a.a) and YBX3 (165–372 a.a)] revealed that myc-tagged HBx interacted exclusively with YBX3 (165–372 a.a) (**Figure 1**). This localized the HBx binding site to amino acids 224–372 of YBX3.

### HBV infection was significantly impaired in YBX3-knockdown/knockout cells

**Figure 2:**
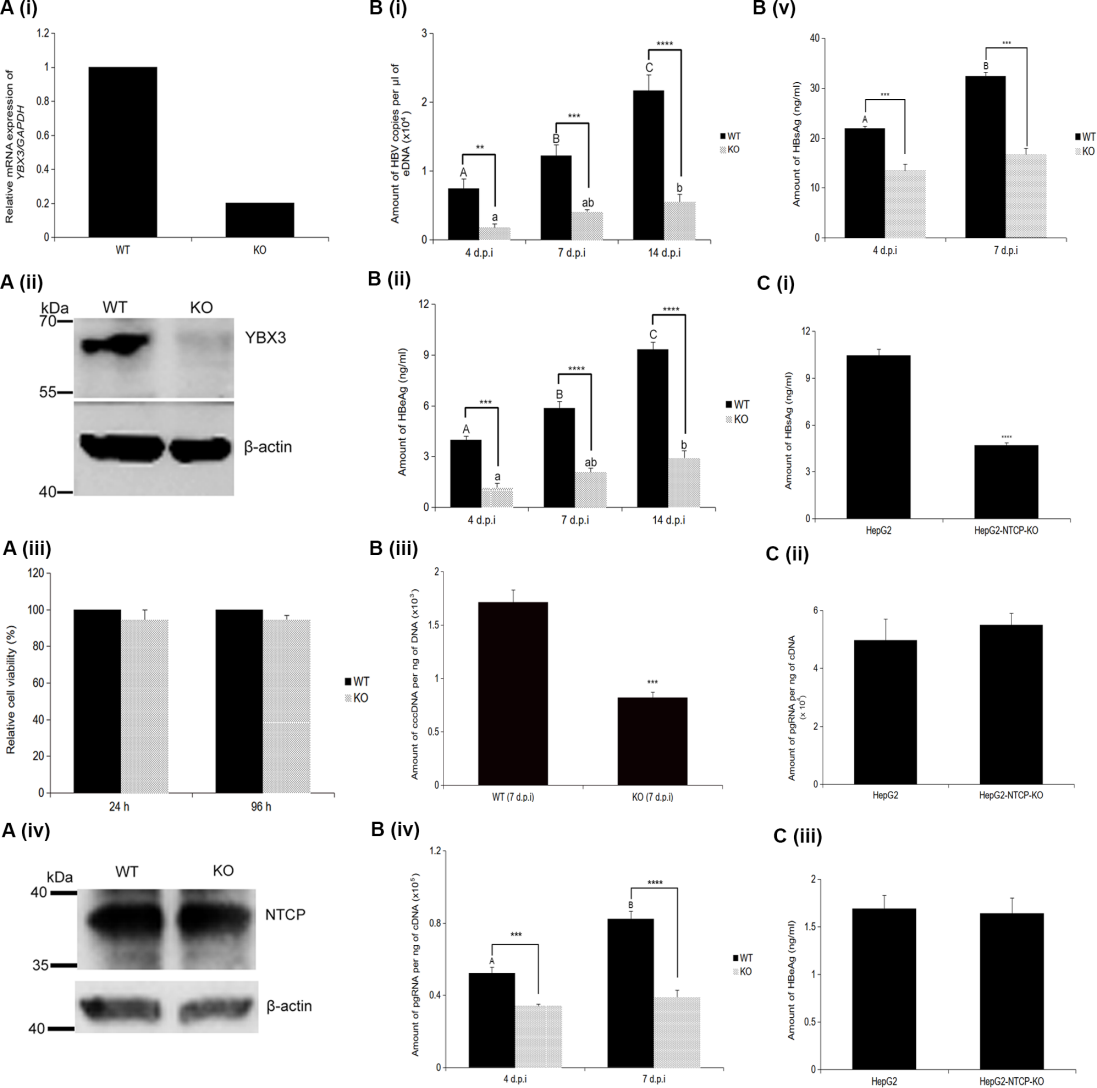
Knockout of YBX3 drastically impaired HBV replication. **(A, i)** Quantification of YBX3 transcript level in both WT and YBX3-KO HepG2-hNTCP cells. (**A, ii**) Western blotting analysis of the protein abundance of YBX3 in WT and YBX3-KO HepG2-hNTCP cells. (**A, iii**) Cell viability of WT and KO cells was measured at 24 h and 96 h. (**A, iv**) Western blotting analysis of the protein abundance of NTCP in WT and YBX3-KO HepG2-hNTCP cells. β-actin served as a loading control. (**B, i**) Quantification of extracellular HBV DNA, (**B, ii**) HBeAg secretion, (**B, iii**) cccDNA, (**B, iv**) pgRNA and (**v**) HBeAg secretion by both WT and YBX3-KO HepG2-hNTCP cells at 4 and/or 7 and/or 14 d.p.i. (**C**, **i**) Quantification of HBsAg secretion, (**C, ii**) pgRNA secretion and (**C, iii**) HBeAg by both parental HepG2 and YBX3-KO HepG2-hNTCP cells at 7 days post-transfection of full HBV genome of genotype **(C)**. Data represent the mean + SEM of three independent biological samples processed at three separate times. Asterisks represent significant p-values, where ∗∗*p* < 0.01, ∗∗∗*p* < 0.001, and ∗∗∗∗*p* < 0.0001. Different letters indicate statistical significance (*p* < 0.05), where groups not sharing a letter are significantly different.

To investigate the role of YBX3 in the HBV life cycle, we first employed transient siRNA-mediated silencing in WT HepG2-hNTCP cells. Successful knockdown was validated by a drastic reduction in *YBX3* mRNA expression (**Supplementary Figures 4, i**). This transient depletion resulted in a significant downregulation in pgRNA (**Supplementary Figures 4, ii**), secreted HBeAg (**Supplementary Figures 4, iii**), and HBsAg (**Supplementary Figure 4, iv**) at both 4 and 6 d.p.i. While siRNA provided initial validation, we utilized CRISPR-Cas9 technology to generate a stable YBX3-KO HepG2-hNTCP line to assess the long-term and permanent effects of YBX3 deficiency. Successful knockout of YBX3 was validated by the drastic reduction in its mRNA expression and protein abundance (**Figures 2A, i, ii**). No significant difference in cell growth was observed between WT and YBX3-KO cells over a 4-day period (**Figures 2A, iii**). Furthermore, NTCP protein levels remained comparable between the two cell lines (**Figures 2A, iv**). This depletion of YBX3 also resulted in a significant downregulation of the level of HBV progeny virus produced (abbreviated as eDNA) and HBeAg levels when compared to wild-type cells at 4, 7 and 14 d.p.i (**Figures 2B, i, ii**). Specifically, in wild-type cells, eDNA and HBeAg levels were significantly increased by 1.77-fold and 1.59-fold, respectively, over a single week. The viral replication in infected YBX3-KO cells was substantially reduced, although there was still a significant increase in both the amount of eDNA and HBeAg when comparing between 4 and 14 d.p.i; however, this overall viral accumulation was markedly reduced as compared to wild-type cells. Analysis of the intracellular viral components at 7 d.p.i. also revealed that the infected YBX3-KO cells contained significantly lower amounts of cccDNA compared to WT cells (~50% reduction) (**Figures 2B, iii**; **Supplementary Figure 5**). This ~50% reduction in the viral template was accompanied by a concomitant decrease in the amount of intracellular pgRNA, which was also reduced approximately by 50% (**Figures 2B, iv**). Additionally, levels of secreted HBsAg were significantly lower in the culture supernatant of YBX3-KO cells at both 4 and 7 d.p.i. compared to WT counterparts (**Figures 2B, v**). To confirm that the deficit in viral replication was independent of viral entry, a plasmid containing 1.2x copy of full-length HBV genome of genotype C (Sugiyama et al., 2006) was transfected into the parental HepG2 and YBX3-KO HepG2-hNTCP cells. In this transfection model, secreted HBsAg levels remained significantly lower in YBX3-KO cells (~55% reduction), mirroring the trend observed from the infection data (**Figures 2C, i**). However, pgRNA and secreted HBeAg levels remained stable and comparable in both cells (**Figures 2C, ii, iii**).

### Ectopic expression of YBX3 partially rescues HBV replication in *YBX3*^(-/-)^ cells

To confirm that the impaired viral replication in *YBX3*^(-/-)^ cells was specifically caused by the loss of YBX3, we performed a functional rescue experiment by transiently overexpressing YBX3. The re-introduction of YBX3 led to a statistically significant increase in several viral replication markers at 5 d.p.i. compared to the EV-transfected knockout cells (**Supplementary Figure 6**). Specifically, extracellular HBV DNA, intracellular pgRNA, and HBeAg levels were significantly higher in cells expressing FLAG-YBX3. However, although these increases were statistically significant, the restoration only reached approximately 20% of the viral levels observed in wild-type cells. Furthermore, in contrast to the other markers, the levels of secreted HBsAg did not show a significant recovery upon the ectopic expression of YBX3.

### Expression of HBx increases nuclear accumulation of endogenous YBX3

**Figure 3:**
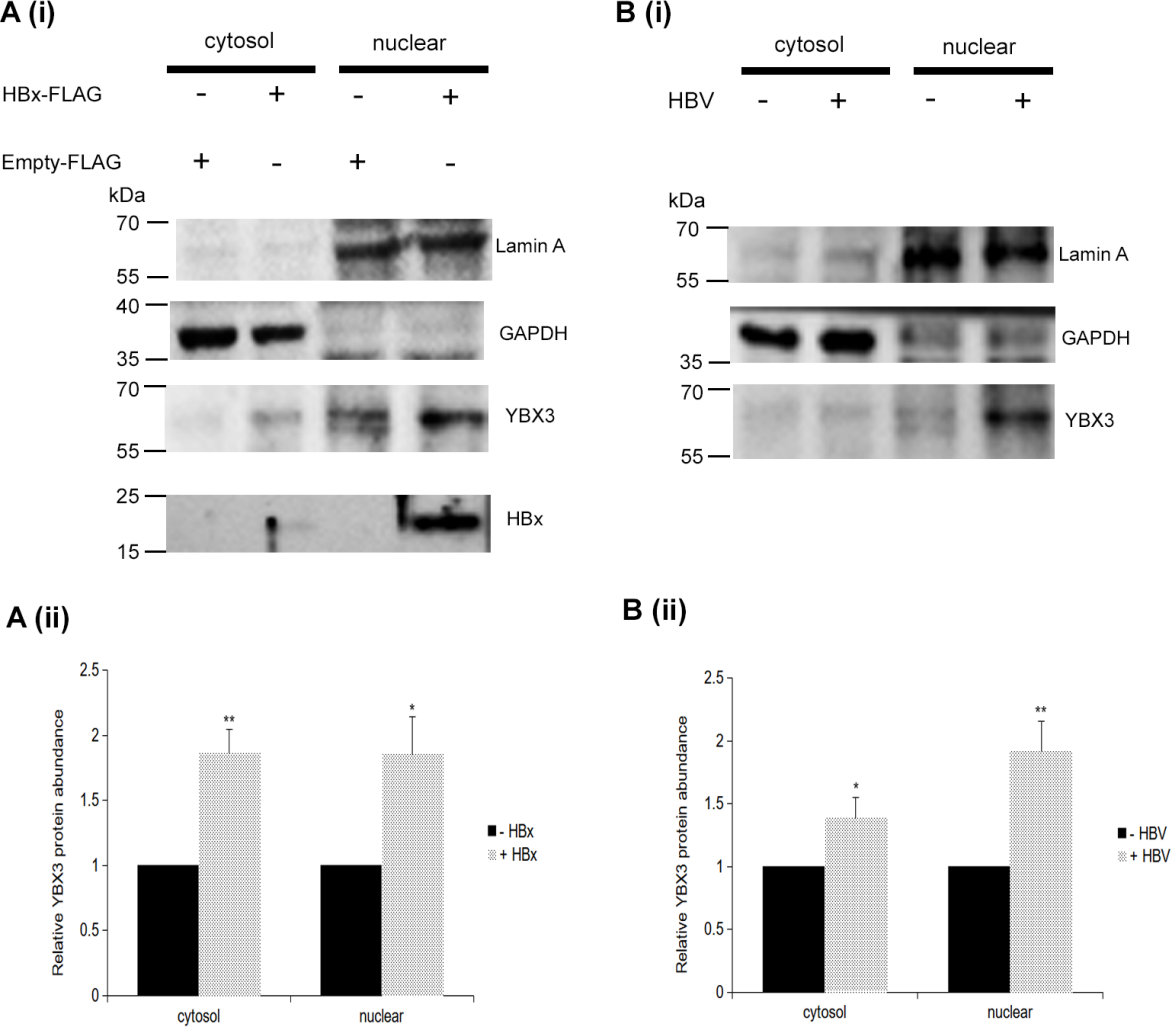
Expression of HBx increases the protein abundance of YBX3 in both subcellular compartments. **(A, i)** HepG2-hNTCP cells were transfected with either an empty vector (control) or FLAG-tagged HBx, and subcellular fractionation was performed subsequently. Fractionated cell lysates were subjected to western blotting analysis to determine the expression of the transfected HBx and the endogenous YBX3 in cytosol and nuclear fractions. Lamin A and GAPDH were used as markers for nuclear and cytosolic fractions, respectively. β-actin served as a loading control. (**A, ii**) Western blotting analysis of YBX3 protein abundance in different compartments of EV-transfected or FLAG-tagged HBx-transfected HepG2-hNTCP cells. (**B, i**) Similar experimental setup as in (**A, i**), but HepG2-hNTCP cells were either mock or infected with HBV for 14 days. (**B, ii**) Western blotting analysis of YBX3 protein abundance in the different compartments of mock or HBV-infected HepG2-hNTCP cells. Data represent the mean + SEM of three independent biological samples processed at three separate times. Asterisks represent significant p-values, where ∗*p* < 0.05 and ∗∗*p* < 0.01.

Subcellular fractionation of HepG2-hNTCP cells revealed that endogenous YBX3 was predominantly localized in the nucleus. Upon transfection with FLAG-tagged HBx, an upregulation in the protein abundance of YBX3 was observed in both the nuclear and cytoplasmic compartments (**Figure 3A**). This increase in YBX3 protein abundance in both compartments was also evident in HBV-infected HepG2-hNTCP cells by 14 d.p.i (**Figure 3B**). Concurrently, subcellular fractionation confirmed that HBx itself was primarily localized to the nucleus of the transfected cells (**Figure 3A**).

### Increased in the mRNA expression and protein abundance of YBX3 during HBV infection

**Figure 4:**
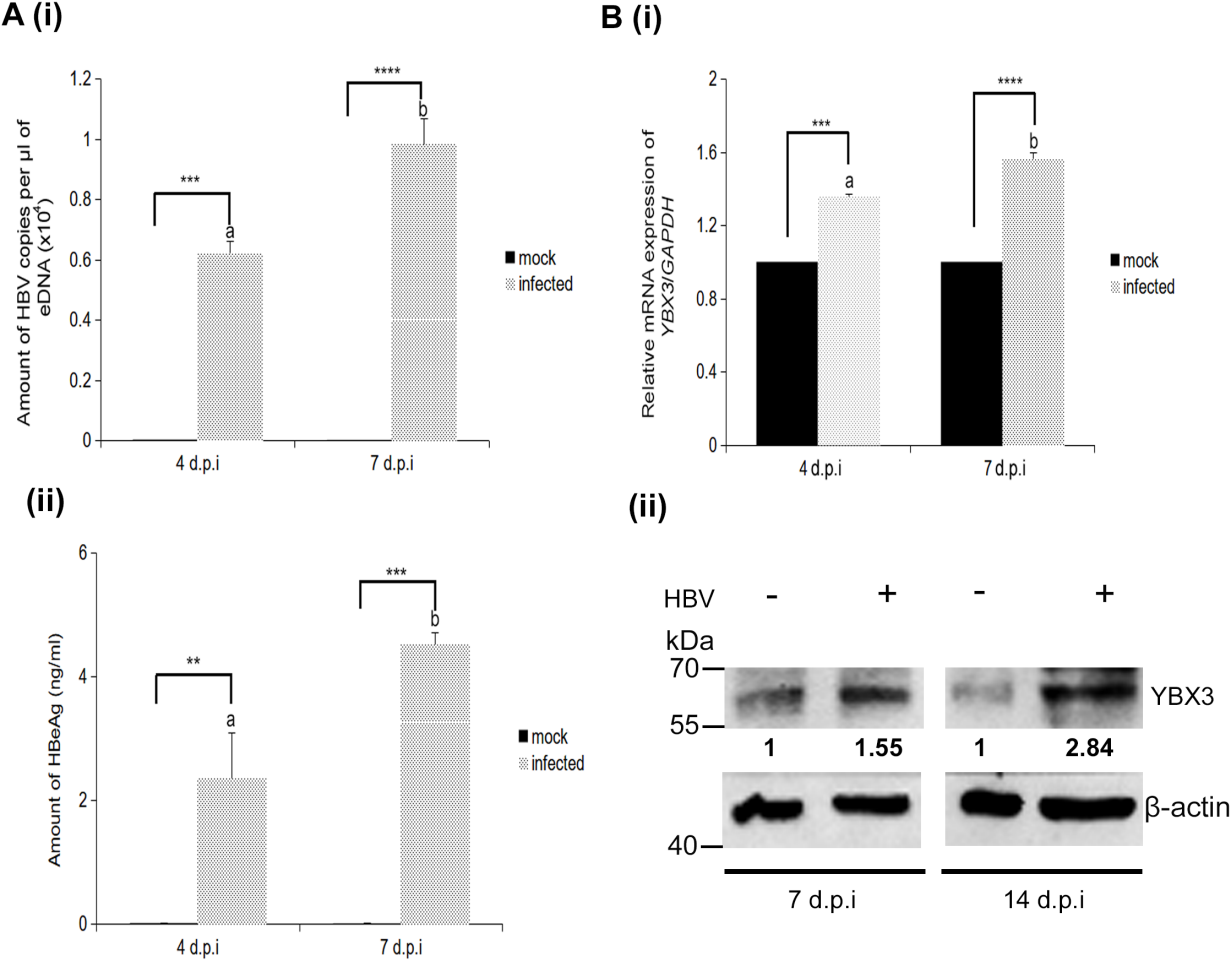
The mRNA and protein expression levels of *YBX3*/YBX3 were significantly upregulated during HBV infection. **(A, i)** Quantification of extracellular HBV DNA and **(A, ii)** HBeAg secretion by wild-type HepG2-hNTCP cells at 4 and 7 d.p.i. (**B, i**) Quantification of mRNA transcript levels of *YBX3* (n=3). (**B, ii**) Western blotting analysis of YBX3 protein abundance in mock or HBV-infected HepG2-hNTCP cells at 7 and 14 d.p.i. Data represent the mean + SEM of three independent biological samples processed at three separate times. Asterisks represent significant p-values, where ∗∗*p* < 0.01, ∗∗∗*p* < 0.001, and ∗∗∗∗*p* < 0.0001. Different letters indicate statistical significance (*p* < 0.05), where groups not sharing a letter are significantly different.

To confirm active viral replication in infected HepG2-hNTPC cells, eDNA was quantified at 4 and 7 d.p.i and was detected at both time-points with significantly higher levels at 7 d.p.i as expected (**Figures 4A**, i). A similar trend was observed for HBeAg secretion, which is an indicator of active viral replication (**Figures 4A**, ii). With successful viral replication confirmed, the mRNA expression levels of *YBX3* in infected cells were compared to mock-infected and upregulation of YBX3 was observed at both time-points (**Figures 4B**, i). To account for the temporal lag between mRNA transcription and protein translation, the protein expression levels of YBX3 was evaluated at 7 and 14 d.p.i, revealing a significant up-regulation in infected cells (**Figure 4B**, ii).

## Discussion

The HBx protein is a multifunctional regulator that manipulates host machinery in both the cytoplasm—such as the CRL4-mediated degradation of Smc5/6—and the nucleus, where it remodels cccDNA chromatin to drive viral transcription (Li et al., 2024; Belloni et al., 2009). While HBx is known to leverage various host factors, many reported interactions lack functional validation. Our study addresses this gap by identifying YBX3 as a novel, functionally essential interactor of HBx.

The YBX family of proteins is named for its members’ ability to bind a specific DNA sequence called the Y box. There are three members—YBX1, YBX2, and YBX3. In general, YBX proteins are highly versatile nucleic acid-binding proteins that act as central hubs in regulating gene expression at multiple levels, from transcription in the nucleus to translation and stability in the cytoplasm and are critical for cellular adaptation and development (Matsumoto and Wolffe, 1998; Mordovkina et al., 2020). As YBX2 is not expressed in the liver, further elaboration on its expression would be omitted. Based on AP-MS screening and co-IP experiments, YBX3 was identified as a novel interactor of HBx. Our mapping studies further revealed that this interaction is mediated by the CTD of YBX3. While CTDs are established mediators of diverse protein interactions (Kohno et al., 2003), structural differences between the CTDs of YBX3 and YBX1 likely account for their functional non-redundancy. Although their Cold Shock Domains (CSDs) are highly conserved (96.2% similarity), their CTDs share only 47.7% similarity. This marked divergence explains why YBX1, despite its known role in HBV replication, does not bind to HBx. The unique evolution of the YBX3 CTD to facilitate this specific recruitment provides a novel mechanistic insight, highlighting a specialized, non-redundant function for YBX3 in the viral-host interactome.

CRISPR/Cas9-mediated knockout of YBX3 in HepG2-hNTCP cells significantly attenuated HBV replication. Crucially, this impairment was not an indirect consequence of reduced receptor expression or compromised cellular health; NTCP levels and cell viability remained stable in YBX3-KO cells, consistent with previous findings that YBX3 depletion does not adversely affect HepG2 cell proliferation (Awad et al., 2023). Following genetic ablation, both viral eDNA, HBeAg secretion were markedly reduced (~60-80%) by day 14 post infection. Consistent with these findings, we quantified pgRNA, cccDNA and HBsAg secretion levels at 7 d.p.i. and found that both were also significantly diminished in YBX3-KO cells. To exclude off-target effects, we performed a genetic rescue experiment. Re-expression of YBX3 in *YBX3*^(-/-)^ cells resulted in a ~20% restoration of pgRNA levels, HBeAg secretion, and eDNA (**Supplementary Figure 6**), providing internally consistent evidence of a functional rescue. The observation that these three markers which depend on the 3.5 kb-transcripts regulated by the core promoter, recovered in tandem suggests that YBX3 specifically facilitates the production or stability of these primary replication templates. In contrast, the lack of a significant increase in HBsAg secretion likely reflects the inherent difficulty in detecting modest changes against the high baseline of HBsAg subviral particles. Crucially, the ~20% recovery of the core-derived markers aligns almost perfectly with the reported 20–25% transfection efficiency of HepG2 cells (Wang et al., 2018). Given the ‘mosaic’ transfection efficiency of HepG2-hNTCP cells and the slow kinetics of the HBV life cycle, the coordinated ~20% increase in pgRNA, HBeAg, and viral eDNA suggests that YBX3 is a requirement for efficient HBV replication. Beyond confirming this specificity, we observed that the impact of YBX3 depletion becomes more pronounced as the infection progresses. The increasing magnitude of this viral suppression in the knockout cells highlights the sustained importance of YBX3 throughout the viral life cycle, particularly as replication ramps up. This correlates with our observation that YBX3 protein abundance naturally increases over a 14-day HBV infection course (**Figures 2B**, **4B**), suggesting that the virus leverages this accumulation to maintain its replication momentum. However, the presence of a residual viral population in the absence of YBX3 suggests that the virus retains a minimal, albeit severely compromised, capacity for replication. While YBX3 depletion profoundly inhibits the virus, a low basal level of replication persisted. This observation is consistent with studies of HBx-deficient HBV, which can establish cccDNA but fails to maintain a productive, self-sustaining infection (Lucifora et al., 2011). Recent evidence indicates that HBx facilitates replication by recruiting m^6^A methyltransferases (METTL3/14) to the HBV minichromosome, a process essential for pgRNA encapsidation and reverse transcription (Kim and Siddiqui, 2022). Therefore, the replication deficit in YBX3-depleted cells likely stems from a disruption of these HBx-mediated functions that rely on YBX3, alongside the inherent basal transcriptional activity of the viral cccDNA. It is unlikely that YBX3 affects the early stage of viral entry as HBsAg secretion remained significantly suppressed in YBX3-KO cells following transfection with 1.2x copy of full-length HBV genome of genotype C. This confirms that YBX3 is required for the intracellular stages of the viral life cycle. Surprisingly, pgRNA and HBeAg levels remained stable. This is not entirely unexpected, as the genotype C basal core promoter contains well-characterized mutations (A1762T/G1764A) which enhance its intrinsic strength (Sugiyama et al., 2006). This resilience suggests that the core promoter in genotype C possesses sufficient transcriptional autonomy to bypass the requirement for YBX3-HBx axis, whereas the S-promoter remains stringently dependent on this axis. Collectively, this evidence establishes YBX3 as a pivotal host factor in promoting HBV replication.

Although YBX3 is a nucleocytoplasmic shuttling protein, subcellular fractionation of HepG2-hNTCP cells consistently revealed its predominant localization within the nucleus. Notably, YBX3 protein abundance increased in both the nuclear and cytoplasmic compartments upon HBx expression, whether via plasmid transfection or physiological infection, underscoring that this upregulation is a direct consequence of HBx presence. Consistent with the known requirements for HBx-mediated replication, we found that both proteins maintain a strong nuclear presence. The critical importance of the nuclear localization of HBx has been well-established; nuclear-targeted HBx can restore the replication of HBx-deficient HBV, whereas cytoplasmically restricted HBx cannot (Keasler et al., 2009). While it remains to be determined whether HBx and YBX3 enter the nucleus as a pre-formed complex or individually, YBX3 is known to facilitate the nuclear translocation of its binding partners (Xie et al., 2023). Whether YBX3 serves as a chaperone for HBx nuclear entry is a compelling area for future investigation. Elucidating the molecular mechanism by which this interaction sustains the nuclear activity of HBx will be essential to fully understanding YBX3’s proviral role.

Our results consistently demonstrated a significant upregulation of YBX3 at both the transcript and protein levels during active HBV infection. This increase occurred concurrently with viral replication; while *YBX3* mRNA levels rose approximately 1.56-fold by 7 d.p.i., protein abundance increased more substantially, reaching ~2.84-fold by 14 d.p.i. (**Figures 2B**, **4B**). These data indicate that HBV infection enhances YBX3 expression through both transcriptional and translational mechanisms. While the precise molecular mechanisms by which HBx drives this increase remain to be fully elucidated, the effect is likely mediated through host transcription factors. For instance, HBx is known to promote the expression of E2F1, a master regulator of cell cycle progression that has been shown to positively regulate the transcription of *YBX3* (Arakawa et al., 2004). It is therefore conceivable that an HBx-E2F1 axis represents a key pathway through which the virus highjacks host machinery to ensure a sufficient supply of YBX3 for sustained replication.

## Conclusion

YBX3 exhibits a functional dichotomy across various viral infections, acting as an antiviral factor against IAV and PEDV (Qin et al., 2020; Wu et al., 2024), while serving a proviral role for SARS-CoV-2 (Zhao et al., 2023). Our study definitively identifies YBX3 as a critical proviral factor for HBV. We demonstrate that YBX3 physically interacts with HBx via its C-terminal domain to facilitate efficient viral replication. Genetic ablation of YBX3 significantly impairs the production of viral markers without compromising host cell viability, a phenotype that is successfully reversed by YBX3 re-expression. These findings establish the YBX3-HBx axis as a vital bottleneck in the HBV life cycle and a promising target for host-directed therapeutic intervention.

## Data Availability

The raw data supporting the conclusions of this article will be made available by the authors, without undue reservation.
