# Supplementary material for: HBx interacts with the host YBX3 protein and up-regulates its expression to mediate efficient Hepatitis B viral replication
Source: Front Cell Infect Microbiol. 2026 May 22;16:1732356. doi: 10.3389/fcimb.2026.1732356 (PMC13237042; doi:10.3389/fcimb.2026.1732356)
Supplement: Supplementary file 1 [file Table1.docx]

**Table S1**. Plasmids used in this study

| **Plasmid name** | **Parent plasmid** | **Insert** | **Source of reference** | **Purpose** |
| --- | --- | --- | --- | --- |
| Empty vector | pXJ40-FLAG | None | This study | Negative control for expression studies |
| Empty vector | pXJ40-myc | None | This study | Negative control for expression studies |
| YBX3 | pcDNA3.1+/C-(K)-DYK | YBX3 (Full length) | From Genscript (OHu03451D) | Co-IP |
| YBX3 (1-223) | pXJ40-FLAG | YBX3 (1-233) | This study | Co-IP |
| YBX3 (161-372) | pXJ40-FLAG | YBX3 (161-273) | This study | Co-IP |
| YBX1 | pcDNA3.1+/C-(K)-DYK | YBX1 (Full length) | From Genscript (OHU23347D) | Co-IP |
| HBx | pxJ40-myc | HBx (Full length) | This study | Co-IP |
| HBx | pxJ40-FLAG | HBx (Full length) | This study | 1) For IP-MS analysis and 2) Overexpression of HBx to study its effect on endogenous YBX3 |
| pUC19_C_JPNAT | pUC19 | 1.2x copies of wild type HBV genome of genotype C | Sugiyama et al., 2006 | To confirm the deficit in viral replication in YBX3-KO cells is independent of viral entry |
| sgRNA1 | pLentiCRISPRv2-GFP | sgRNA targeting exon 1 of *YBX3* | Addgene #82416 | CRISPR-Cas9-mediated knockout |
| sgRNA2 | pLentiCRISPRv2-GFP | sgRNA targeting exon 10 of *YBX3* | Addgene #82416 | CRISPR-Cas9-mediated knockout |
| pRRE | pMD | HIV-1 Gag/Pol, RRE | Addgene #12251 | Lentiviral packaging plasmid |
| pRSV-Rev | N/A | HIV-Rev | Addgene #12253 | Lentiviral packaging plasmid |
| pMD2.G | N/A | VSV-G envelope glycoprotein | Addgene #12259 | Lentiviral packaging plasmid |

**Table S2**. Primary antibodies used in this study

| **Antibodies** | **Supplier** | **Cat no.** | **Clone no.** |
| --- | --- | --- | --- |
| Lamin A/C | Santa Cruz | sc-20681 | H-110 |
| Mouse c-myc | Santa Cruz | sc-40 | 9E10 |
| Anti-FLAG | Sigma | F7425 | N/A |
| Zonab (YBX3) | Thermo Fisher | A303-070A | N/A |
| Anti-GAPDH | Sigma | ZRB374 | 10B13 |
| Anti-β-Actin | Sigma | A5441 | N/A |
| Anti-NTCP | Thermo Fisher | PA5-25614 | N/A |

**Table S3**. qPCR primers used in this study

| **Gene** | **Forward primer** | **Reverse primer** |
| --- | --- | --- |
| *YBX3* | 5′-TGGCCCGGATGGAGTTCCTG-3′ | 5′-GGCAGGTCGTGGGCGA-3′ |
| *YBX1* | 5′-TGCAGCAGACCGTAACCATT-3′ | 5′-TGGATCGGCTGCTTTTGTC-3′ |
| *GAPDH* | 5′-GCCATCAATGACCCCTTCATT-3′ | 5′-TCTCGCTCCTGGAAGATGG-3′ |

**Table S4.** Full list of host proteins interacting with HBx identified by IP-MS. These potential host interactors were selected based on positive log-fold change (logFC > 0) and a p-value less than 0.05 (*p*<0.05), indicating statistically significant enrichment. Percentage coverage was calculated based on the ratio of the total number of amino acid residues identified by the peptides to the total number of amino acid residues in the full-length protein.

| **Protein (Abbreviation)** | **Percentage coverage (%)** |
| --- | --- |
| Heat shock cognate 71 kDa (HSPA8) | 62.50 |
| Reptin (RUVBL2) | 43.19 |
| BAG family molecular chaperone regulator 2 (BAG2) | 34.60 |
| Y-box -binding protein 3 (YBX3) | 33.60 |
| Signal recognition particle receptor subunit beta (SRPRB) | 31.37 |
| U2 small nuclear ribonucleoprotein B (SNRPB) | 23.11 |
| 40S ribosomal protein S15 (RPS15) | 21.71 |
| Small ribosomal subunit protein eS26 (RPS26) | 20.87 |
| Cytoplasmic dynein 1 intermediate chain 2 (DYNC1I2) | 16.61 |
| Heat shock protein 105 kDa (HSPH1) | 16.20 |
| Serotransferrin (TF) | 15.61 |
| Galactokinase (GALK1) | 14.80 |
| Nuclear cap-binding protein subunit 2 (NCBP2) | 12.18 |
| Interferon gamma receptor 1 (IFNGR1) | 11.25 |
| Na(+)/H(+) exchange regulatory cofactor NHE-RF1 (NHERF1) | 11.17 |
| Glucose-6-phosphate 1-dehydrogenase (G6PD) | 9.90 |
| Large ribosomal subunit protein eL36 (RPL36) | 9.52 |
| Large ribosomal subunit protein eL29 (RPL29) | 9.43 |
| cAMP-dependent protein kinase type II-alpha regulatory subunit (PRKAR2A) | 8.42 |
| Aspartyl aminopeptidase (DNPEP) | 7.71 |
| F-box-like/WD repeat-containing protein TBL1XR1 (TBL1XR1) | 7.39 |
| Armadillo repeat-containing X-linked protein 3 (ARMCX3) | 7.12 |
| Guanine nucleotide-binding protein-like 3 (GNL3) | 7.10 |
| Calcineurin B homologous protein 1 (CHP1) | 6.15 |
| Large proline-rich protein BAG6 (BAG6) | 4.48 |
| 6-phosphogluconate dehydrogenase, decarboxylating (PGD) | 4.35 |
| Copine-1 (CPNE1) | 3.72 |
| Tumor necrosis factor receptor superfamily member 10B (TNFRSF10B) | 3.41 |
| Estradiol 17-beta-dehydrogenase (HSD17B1) | 3.35 |
| Zinc finger protein 207 (ZNF207) | 2.72 |
| Fibroblast growth factor receptor 4 (FGFR4) | 2.62 |
| Lysosome-associated membrane glycoprotein 2 (LAMP2) | 1.95 |
| Nondiscriminating glutamyl-tRNA synthetase EARS2, mitochondrial (EARS2) | 1.34 |
| ATP-dependent RNA helicase DDX18 (DDX18) | 1.34 |
| Protein transport protein Sec24C (SEC24C) | 1.19 |
| TRPM8 channel-associated factor 1 (TCAF1) | 1.09 |
| Activating molecule in BECN1-regulated autophagy protein 1 (AMBRA1) | 1.08 |
| Neuroblast differentiation-associated protein AHNAK (AHNAK) | 0.20 |
