# Supplementary material for: HBx interacts with the host YBX3 protein and up-regulates its expression to mediate efficient Hepatitis B viral replication
Source: Front Cell Infect Microbiol. 2026 May 22;16:1732356. doi: 10.3389/fcimb.2026.1732356 (PMC13237042; doi:10.3389/fcimb.2026.1732356)

**Fig. S1 (A)** Uncropped blots of YBX3 and β-actin proteins in WT and YBX3-KO HepG2-hNTCP cells. **(B)** Uncropped blots of NTCP and β-actin proteins in WT and YBX3-KO HepG2-hNTCP cells.


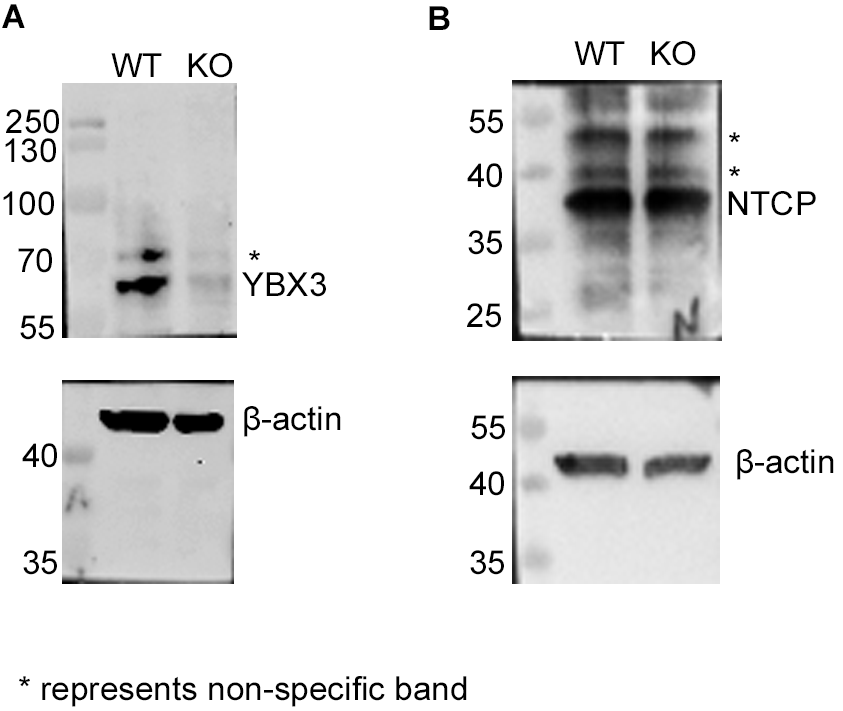


**Fig. S2** Uncropped blots of Lamin A, GAPDH, YBX3, β-actin and HBx proteins in the cytosol and nuclear fraction of (**A**) mock/transfected and (**B**) mock/infected HepG2-hNTCP cells for three independent experiments.


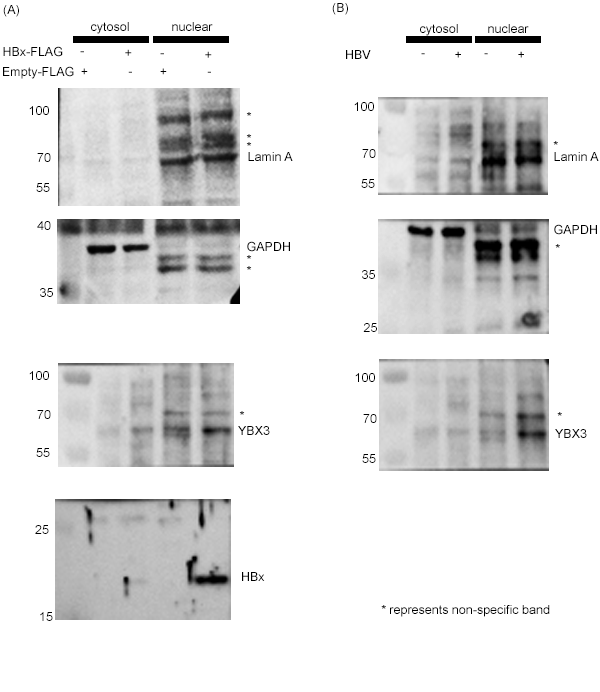


**Fig. S3** Uncropped blots of YBX3 and β-actin proteins in mock and HBV-infected HepG2-hNTCP cells at 7 and 14 d.p.i.


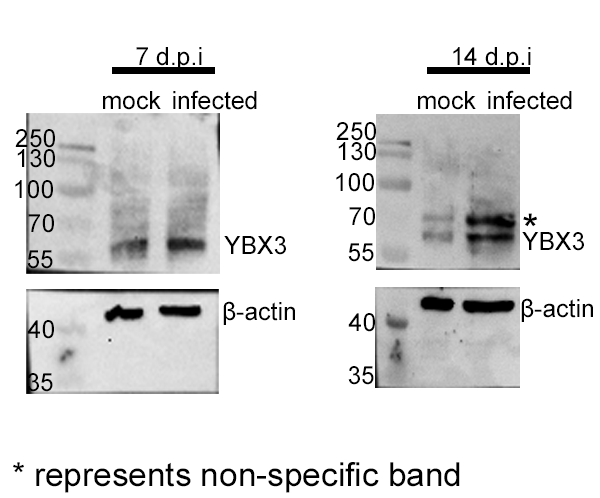


**Fig. S4 Infected YBX3-knockdown cells demonstrated a drastic reduction in viral replication.** YBX3 expression in WT HepG2-hNTCP cells were transiently silenced 48 h prior to HBV infection. (**i**) The silencing of YBX3 expression was validated through qPCR. Quantification of (**ii**) intracellular pgRNA, (**iii**) HBeAg and (**iv**) HBsAg secretion at 4 or 6 d.p.i. Data represent the mean + SEM of three independent biological samples processed at three separate times. Asterisks represent significant p-values, where ∗∗*p*<0.01, ∗∗∗*p*<0.001, and ∗∗∗∗ *p*<0.001


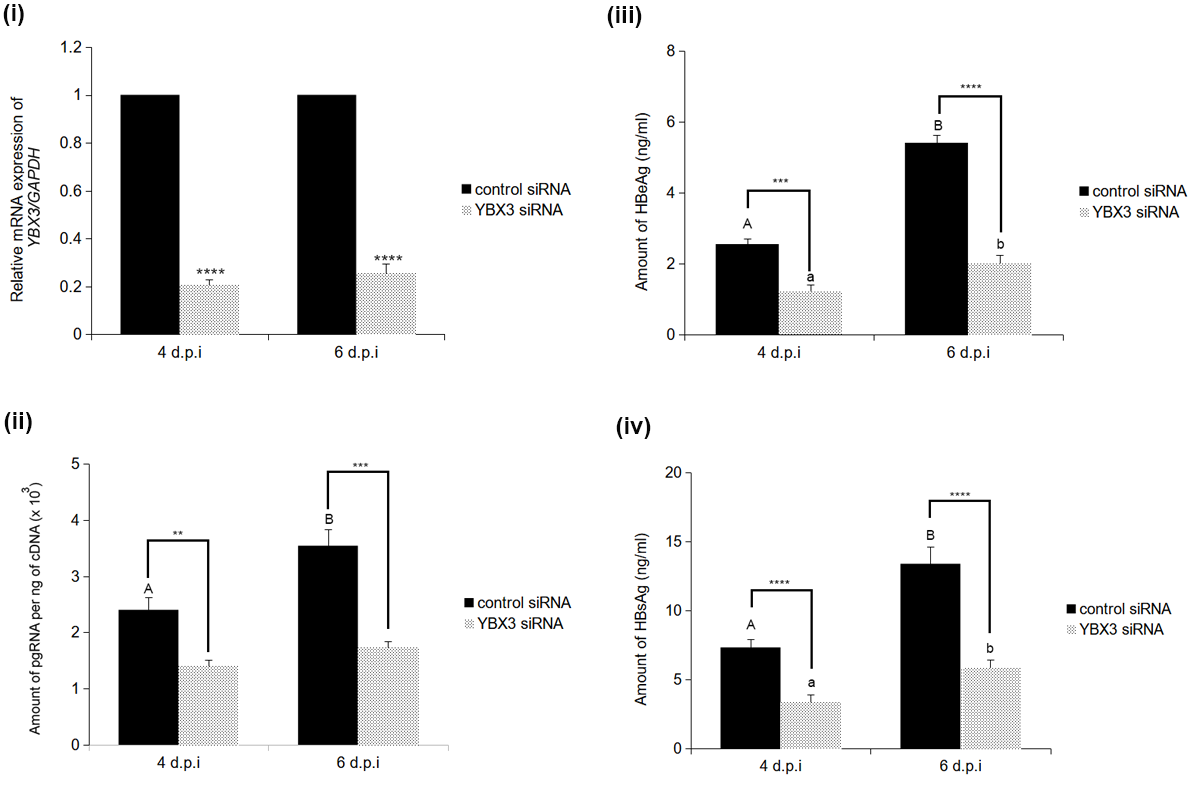


**Fig. S5** Quantification of cccDNA at 7 d.p.i was validated using gap-spanning primers.


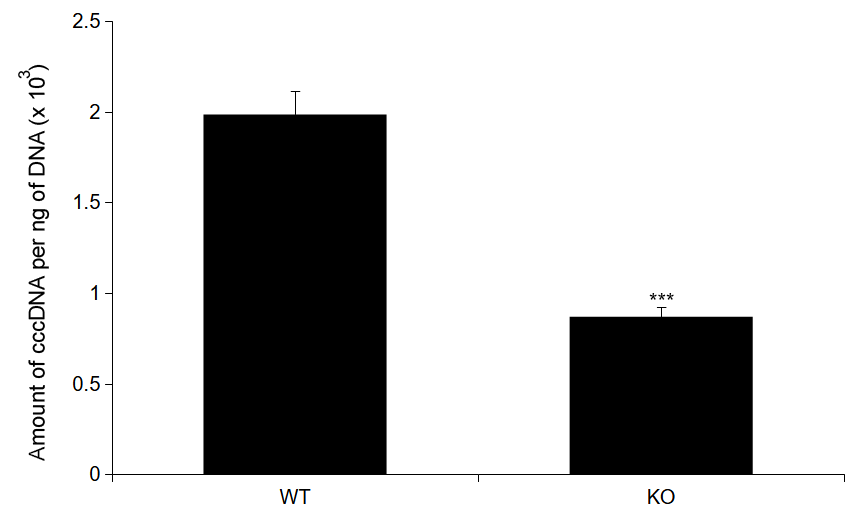


**Fig. S6 Re-expression of YBX3 partially restores viral replication in YBX3-KO cells.** *YBX3*^(-/-)^ HepG2-hNTCP cells were transfected with either a FLAG-YBX3 (+ YBX3) or an empty vector (EV; - YBX3) 24 h prior to HBV infection. The presence of YBX3 in YBX3-transfected cells was validated at 6 days post-transfection (**i**). Quantification of (**ii**) extracellular HBV DNA (eDNA), (**iii**) intracellular pgRNA, (**iv**) HBeAg and (**v**) HBsAg secretion at 5 d.p.i (6 days post-transfection). Data represent the mean + SEM of three independent biological samples processed at three separate times. Asterisks represent significant p-values, where ∗∗*p*<0.01, and ∗∗∗*p*<0.001.


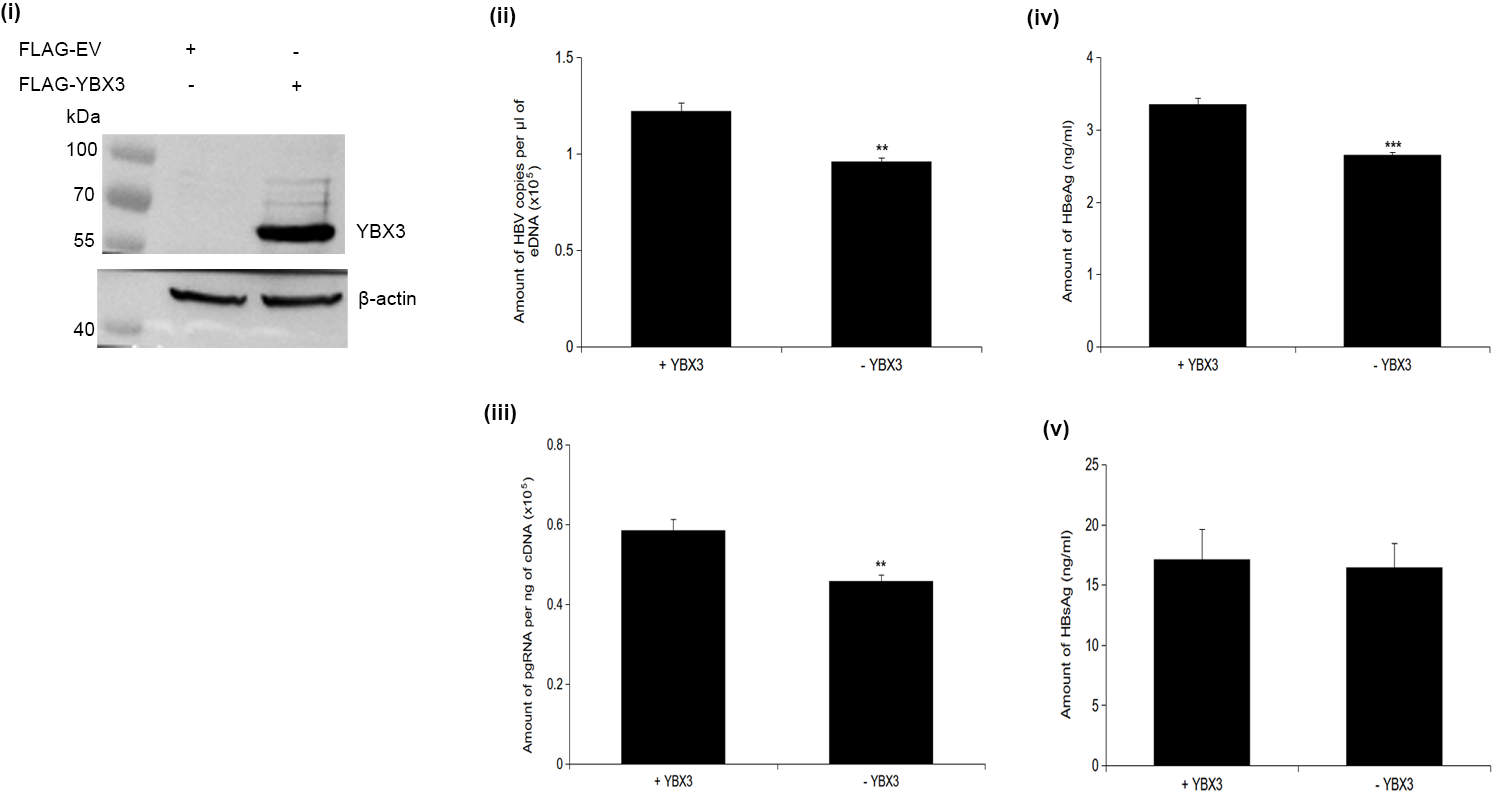

Supplement: Supplementary file 2 [file Table2.docx]
